# Protein translational control and its contribution to oncogenesis revealed by computational methods

**DOI:** 10.1186/1471-2105-16-S2-A6

**Published:** 2015-01-28

**Authors:** Yi Zhong, Phillip Drewe, Andrew L Wolfe, Kamini Singh, Hans-Guido Wendel, Gunnar Rätsch

**Affiliations:** 1 Memorial Sloan Kettering Cancer Center, 1275 York avenue, New York, NY 10065, USA

## Background

Protein translation is a fundamental biochemical process and the regulation of this process in response to a variety of changes has been demonstrated to play a key role in cellular functional activity. Recently, the translational control of oncogenes is implicated in many cancers [1].

## Results

**Figure 1:**
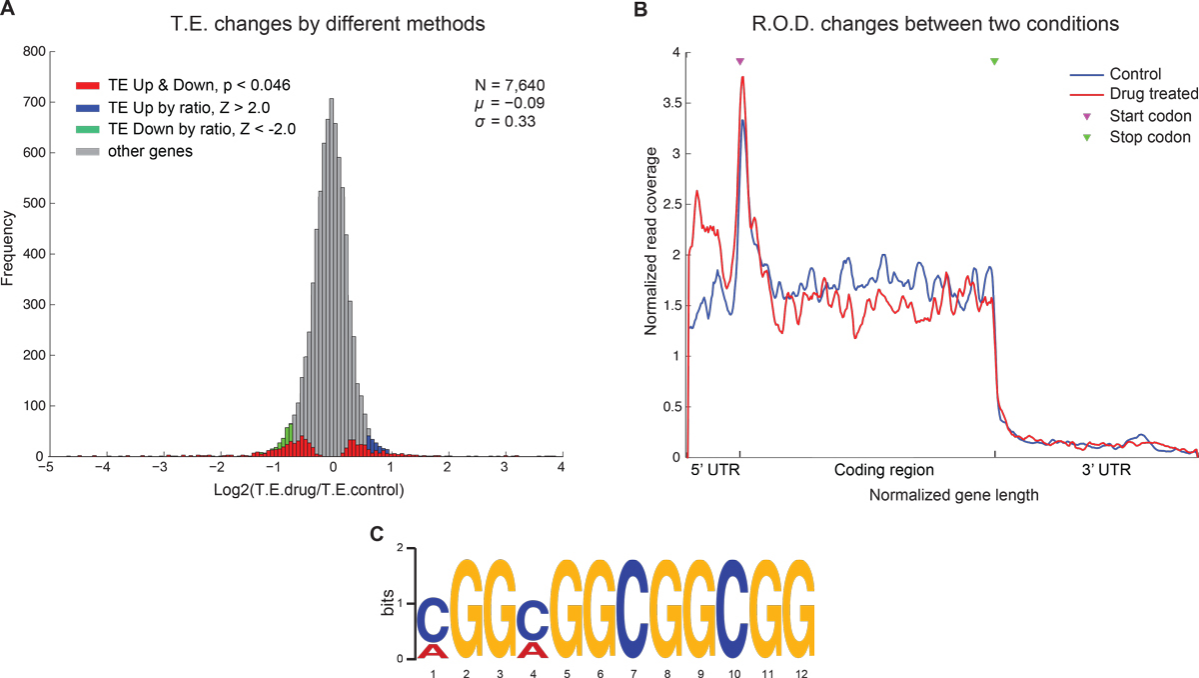
**Computational methods revealed drug effects on protein translation**. **A **Histogram of the ratio of TE in control and drug-treated samples. Red: genes with significant TE down and up regulation were identified based on the read count of Ribo-seq data. Blue and green: TE up and down genes defined by |Z score| > 2 as often used in other analyses. **B **Averaged distribution profile of ribosome occupancy of 62 drug-sensitive genes. Ribosome footprint coverages and transcript lengths were normalized. **C **Twelve-nucleotide motif that is highly enriched in 5' UTR of TE down and ROD positive genes. We suggested that the GQ structure is responsible for ribosome stalling in the 5' UTR [2].

We recently reported a translation initiation factor eIF4A RNA helicase-dependent mechanism of translational control that contributes to oncogenesis and underlies the anticancer effects of drug silvestrol [2]. Inhibition of eIF4A with silvestrol has powerful therapeutic effects *in vitro *and *in vivo*. In this study, we developed novel computational tools, specifically be tailored to study high throughput ribosome footprint data (Ribo-seq) [3], to identify the genes featuring either one of the two changes between two experiment conditions: 1) translational efficiency (TE), and 2) ribosome occupancy distribution profile (ROD) on mRNA. In the parametric test of TE, we take RNA abundance and ribosome occupancy density into account in order to expeditiously identify differential translation efficiency. Whereas the non-parametric test of ROD [4] aims to identify differential occupancy profiles, such as ribosome stalling at specific sites even if overall translation efficiency remain unchanged. Using transcriptome-scale ribosome footprinting data of leukemia cell line, we defined drug-sensitive genes showing both decrease of translational efficiency (Figure 1A) and accumulation of ribosome occupancy at 5'UTR (Figure 1B). Among the most eIF4A-dependent transcripts are a number of oncogenes, super-enhancer associated transcription factors and epigenetic regulators.

## Conclusions

Computational and statistical methodologies facilitate the discovery of the hallmark of eIF4A-dependent transcripts, namely 5'UTR sequence harbors the 12-mer guanine quartet (CGG)_4 _motif associated with RNA G-quadruplex (GQ) structures (Figure 1C). Our novel computational tools provide a fast, accurate solution to gain biological insights from Ribo-seq and RNA-seq data.
